# Development of Magnesium Phosphate Cement Based on Low-Grade MgO

**DOI:** 10.3390/ma18061198

**Published:** 2025-03-07

**Authors:** Ines Garcia-Lodeiro, Salma Chhaiba, Nuria Husillos-Rodriguez, Ángel Palomo, Hajime Kinoshita

**Affiliations:** 1 Eduardo Torroja Institute for Construction Science (IETcc-CSIC), 28033 Madrid, Spain; salma.chhaiba@ietcc.csic.es (S.C.); nuria.husillos@ietcc.csic.es (N.H.-R.); palomo@ietcc.csic.es (Á.P.); 2 Department of Material Science and Engineering, University of Sheffield, Sheffield S10 2TN, UK; h.kinoshita@sheffield.ac.uk

**Keywords:** low-grade MgO, magnesium phosphate cements, K-struvite, volume stability, M/P ratio

## Abstract

Magnesium phosphate cements (MPCs) are a class of inorganic cements that have gained significant attention in recent years due to their exceptional properties and diverse applications in the construction and engineering sectors, particularly in the confinement of radioactive waste. These cements set and harden through an acid–base reaction between a magnesium source (usually dead-burnt magnesia) and a phosphate source (e.g., KH_2_PO_4_). The dead-burnt MgO (DBM) used is typically obtained by calcining pure MgCO_3_ at temperatures between 1600 and 2000 °C. The present work explores the possibility of using low-grade magnesia (≈58% MgO), a secondary waste product generated during the calcination of magnesite for sintered MgO production. Low-grade magnesia is a by-product from the calcination process of natural magnesite. In this manner, the cost of the products could be substantially diminished, and the cementitious system obtained would be a competitive alternative while enhancing sustainability criteria and recyclability. This paper also evaluates the effect of the M/P ratio and curing conditions (especially relative humidity) on the mechanical, microstructural, and mineralogical development of these cements over a period of up to one year. Results indicate that low-grade MgO is suitable for the preparation of magnesium potassium phosphate cements (MKPCs). The presence of minor phases in the low-grade MgO does not affect the precipitation of K-struvite (KMgPO_4_·6H_2_O). Moreover, the development of these cements is highly dependent on both the M/P molar ratio and the RH. Systems prepared with an M/P ratio of 3 demonstrated good compressive strengths, low total porosity, and stable mineralogy, which are essential parameters for any cementitious matrix that aims to be considered as a potential confiner of radioactive waste.

## 1. Introduction

Magnesium phosphate cements (MPCs) represent a class of inorganic cements that have garnered considerable interest in recent years for their exceptional properties and diverse applications in the construction and engineering sectors; one of their most interesting applications is in the confinement of radioactive waste [1,2,3]. Low- and intermediate-level nuclear wastes (LLW and ILW) are commonly conditioned through encapsulation, a process that immobilizes radioactive materials within cementitious matrices. Conventional Portland cement-based materials are widely used for this purpose [4,5,6,7]. However, Portland-based cements set and harden through hydration reactions, making the water content within these cementitious matrices a crucial factor in their performance. The presence of water plays a key role in the hydration process, influencing the microstructure, mechanical strength, and long-term durability of the solidified waste form. One of the inherent risks associated with encapsulating radioactive wastes in such systems is the potential risk of radiolysis of the water due to the ionizing radiation emitted by radioactive isotopes present in the waste. Radiolysis can lead to the decomposition of water molecules, resulting in the emission of H_2_ gas, with the consequent risk of expansion, cracking, explosion, and even fire, reducing the overall safety of the waste forms. The accumulation of these gases within the cement matrix can cause an internal pressure buildup, potentially leading to microcracking, reduced structural integrity, and the release of radioactive contaminants over time [8,9]. With water playing a big role in the disadvantages of using Portland cement to encapsulate radioactive wastes, the logical step to take is to consider using alternative cements. The alternative cementitious binders under investigation in the open literature for potential encapsulation of reactive wastes include calcium sulphoaluminate cements (CŠAs) [10], alkali-activated cements (or geopolymers) [11], phosphate-modified calcium aluminate cements (CAPs) [12,13,14,15], and magnesium phosphate cements (MPCs) [1,2,3]. The focus of the present investigation is MPCs, one of the less studied but most interesting systems. MPCs, unlike Portland cement, set and harden via acid–base reaction between the MgO used as a base and the phosphates used as an acid. Because of this different mechanism of solidification, phosphate-based systems such as MPCs can generate cementitious products with a reduced water content [16], which can be beneficial to avoid hydrogen gas generation associated with the radiolysis of water induced by radioactive waste components. The solidification mechanism for MPCs has been investigated and can be generalized by Reaction 1:

MgO + XH_2_PO_4_ + 5H_2_O → XMgPO_4_·6H_2_O (R1)

where X is commonly NH^4+^ or K^+^, forming a crystalline struvite (NH_4_MgPO_4_·6H_2_O) when NH_4_PO_4_ is used and K-struvite (MgKPO_4_·6H_2_O) with KH_2_PO_4_. The struvite mineral family is known to accept a wide range of substituents within the M1M2A·6H_2_O structure [16]. These include substitutions of monovalent cations (NH^4+^, K^+^, Rb^+^, Cs^+^, Tl^+^) in the M1 site, divalent cations (Mg^2+^, Ni^2+^, Zn^2+^, Co^2+^, Cd^2+^, Cr^3+^, Mn^2+^, VO^2+^) in the M2 site, and trivalent oxyanions (PO_4_^3−^, AsO_4_^3−^) in the A site.

Although MPC binders are relatively less well understood, they can be a good alternative when conventional Portland cement-based materials are not applicable; this is due to the MPC binders’ unique properties, which include near-neutral pH [17], low water demand [18], low drying shrinkage [19], rapid setting [20], and high early compressive strength [21]. In these systems, the water is chemically bound within the K-struvite crystal structure [21], preventing it from enhancing the transportation of ions and their reactions, such as the corrosion of any reactive metal surface present. However, a notable disadvantage is their high cost. Dead-burnt MgO (DBM), used as the basic precursor, is obtained usually from a calcination process of pure MgCO_3_ at temperatures of 1600–2000 °C, which involves a very high energy cost, not to mention additional costs, such as for separating the MgCO_3_ from impurities (i.e., SiO_2_ and Fe_2_O_3_) that may be in the quarry. What is more, MPCs are relatively new cements (the scientific literature on the chemical specifics of MPCs did not emerge until the early 1980s), and therefore, additional studies are still required to understand their behavior with different parameters, such as the M/P ratio and curing conditions. The existing literature on these aspects is scarce and sometimes contradictory. It should also be noted that, practically in all cases, DBM has been the sole material used as the MgO source. Only a few studies in the literature have explored the use of low-grade MgO for the production of MKPC cements, and these are for purposes other than radioactive waste confinement. Maldonado-Alameda et al. [22] developed thermal sustainable magnesium phosphate cement (TS-MPC) as an eco-friendly alternative to ordinary Portland cement (PC) using a low-grade MgO by-product; they incorporated microencapsulated phase-change materials (MPCMs) and an air-entraining additive (AEA) to enhance thermal performance. The study found that TS-MPC reduces thermal fluctuations, lowers HVAC energy use, and decreases CO_2_ emissions in buildings. Niubó et al. [23] aimed to enhance the thermal performance of sustainable magnesium phosphate cement (sust-MPC) by developing a formulation using low-grade MgO and optimizing its porosity and thermal conductivity for improved insulation properties. Opara et al. [24] explored KOH-activated softwood lignin biochar (5 wt.%) in MKPC, improving mechanical properties and sustainability for sustainable precast applications. However, as noted above, there is no work in the literature where low-grade MgO has been used to design a material, especially for radioactive waste confinement. Considering the limitations mentioned above, the main aim of the current work is to explore the possibility of using low-grade magnesia (MgO ~58%), a secondary product obtained from the production of sintered MgO via the calcination of natural MgCO_3_, for the preparation of MKPCs. This would significantly help to reduce the costs for the production of MPCs.

This research focused not only on studying the suitability of this low-grade MgO to develop MKPCs but also on analyzing the effect of curing conditions, and in particular the relative humidity and the M/P molar ratio, on the mechanical properties, mineralogy, microstructure, and volume stability of this alternative cementitious binder. All of these properties will be key parameters in establishing the waste acceptability criteria (WAC) of the waste form, and therefore, it will be essential to develop reference systems with good mechanical strengths, low initial porosity, good volume stability, and a defined mineralogy.

## 2. Materials and Methods

### 2.1. Materials

**Figure 1:**
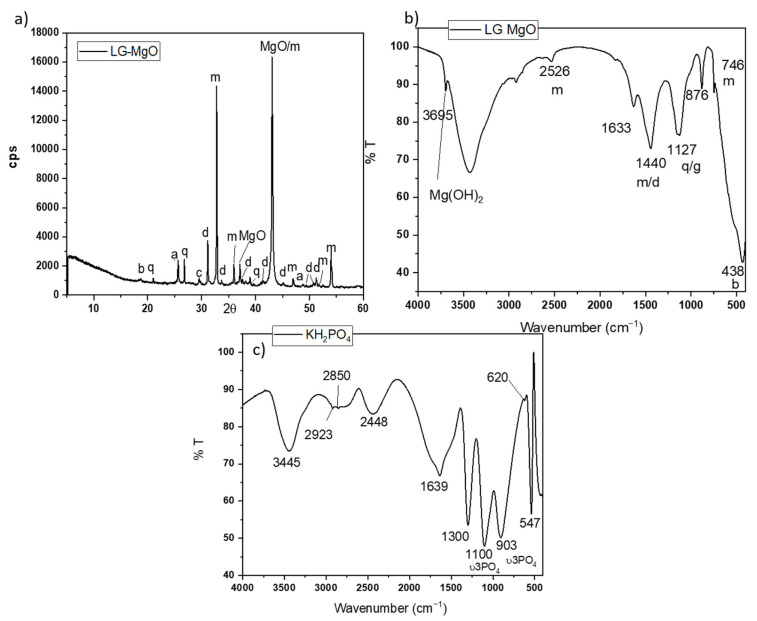
(**a**) XRD patterns and (**b**) FTIR spectrum of low-grade MgO (LG-MgO), (**c**) FTIR spectrum of KH_2_PO_4_. Legend: b: brucite (Mg(OH)_2_) (PDF 74-2220); q: quartz (SiO_2_) (PDF 65-0466); a: anhydrite (CaSO_4_) (PDF 37-1496), c: calcite (CaCO_3_) (PDF 05-0586), d: dolomite (MgCa(CO_3_)) (PDF05-0622), m: magnesite, MgCO_3_ (PDF 08-0479).

A low-grade MgO, provided by MAGNESITAS NAVARRAS S.A (Zubiri, Spain), was used as a source of MgO. As the magnesite (MgCO_3_) enters the calcining kiln for the production of dead-burnt magnesia (DBM) (calcining temperature above 1600 °C), a high-temperature air stream circulates in countercurrent, carrying a large amount of dust resulting from the mechanical attrition of the particulate material inside the kiln. The flue gases are fed to a scrubbing system before being emitted into the atmosphere, where the flying particles are retained, initially in a cyclone system and subsequently in a bag filter. The dust retained by the bag filters and cyclones is the so-called cyclone dust (PC), which is the by-product of the calcination process of natural magnesite (referred to as low-grade MgO). Due to its origin and production process, this by-product is mostly composed of MgO. However, it also contains significant amounts of uncalcined MgCO_3_ and dolomite (MgCa(CO_3_)_2_), as confirmed by XRD analysis. Its X-ray diffraction (XRD) pattern is shown in Figure 1a. Rietveld analysis (RWp 7.62) revealed that the main crystalline phase was periclase (MgO 57.62%) with a significant presence of magnesite (MgCO_3_ 25.77%). Some minor components, such as hematite (Fe_2_O_3_ 2.30%), calcite (CaCO_3_ 0.70%), dolomite (MgCa(CO_3_)_2_ 5.41%), anhydrite (CaSO_4_ 3.97%), quartz (SiO_2_, 1.93%), and brucite (Mg(OH)_2_ 2.67%), were also detected.

The Fourier transfer infrared (FTIR) spectrum (in Figure 1b) shows bands associated with the vibration modes of the phases identified by XRD: the bands at 1440, 876, and 746 cm^−1^ are associated with different C-O vibration modes in magnesite and dolomite [25,26]; the band around 1127 cm^−1^ corresponds to the overlapping of the Si-O stretching vibrations of quartz and S-O vibrations of anhydrite; and a sharp peak around 3695 cm^−1^ [26] corresponds with the O-H asymmetrical stretching vibrations of brucite (which is likely the result of the slight hydration of MgO during storage). The particle size (measured by laser granulometry) shows a D10, D50, and D90 of 6.07 μm, 23.2 μm, and 59.90 μm, respectively, and the surface area (BET analysis) is 186.06 m^2^/g.

As source of phosphate, a KH_2_PO_4_ soluble salt (sold as fertilizer) with a purity > 98 wt.% was used. The FTIR spectrum in Figure 1c shows several bands located in different ranges. Bands in the range of 1300–950 cm^−1^ are associated with P-O stretching vibrations, while those in the range of 640–400 cm^−1^ are assigned to O-P-O bending vibrations [27,28]. A laboratory-grade boric acid (H_3_BO_3_) with a purity >99.5 wt. % was added to the systems in the present investigation as a setting retarder [29].

### 2.2. Preparation of MKPC Pastes and Mortars

Table 1 shows the formulation of the MKPC pastes and mortars prepared. Each series was prepared with a constant water/binder (w/b) ratio but with different MgO/KH_2_PO_4_ (M/P) molar ratios. For the preparation of pastes, the w/b ratio was maintained at 0.25, while the boric acid/(KH_2_PO_4_ +MgO) mass ratio was fixed at 0.025. Boric acid was pre-dissolved in water and added to the binder (low-grade MgO + KH_2_PO_4_). The workability of the pastes was, in all cases, similar. The systems were mixed using a high-shear mixer at 1600 rpm for 3 min. With the pastes, prismatic specimens (1 × 1 × 6 cm^3^) were prepared and cured under two types of curing conditions: (i) in the climatic chamber (CC) at 21 ± 2 °C and 99 ± 5% relative humidity (RH) and (ii) and in the laboratory (LAB) at 21 ± 2 °C and 52 ± 5% RH. The mechanical strength of the paste specimens was tested at various ages: 1 day, 7 days, 28 days, 90 days, 180 days, and 365 days. Additionally, selected samples were characterized from mineralogical, nanostructural, and microstructural perspectives at these intervals.

Additionally, mortars (prismatic specimens of 2.5 × 2.5 × 28.5 cm^3^) of MKPCs with a sand/binder mass ratio of 1 and water/binder mass ratio of 0.3 and with different M/P molar ratios (1, 2, 3, and 4) were made. For the preparation of these mortars, normalized quartz sand was used (according to DIN EN 196-1 [30], standard sand has particle sizes ranging from 0.08 to 2.00 mm). These mortars were cured for 24 h at 21 °C and 99% RH, then placed in two environments: climatic chamber (21 °C, 99% RH) and laboratory (21 °C, 52% RH). The changes in the volume stability of the prisms were studied and periodically measured over a period of up to 365 days.

### 2.3. Experimental Methods

The compressive strengths were determined in pastes after 1, 7, 28, 90, 180, and 365 days. Six replicates were tested for each on an Ibertest Autest 200/10-SW (Madrid, Spain) test frame, and the average values and standard deviation were determined. Fragments recovered from the strength testing of the hardened paste samples after 28 days were kept immersed in isopropanol for 2 days to arrest the reactions and then in a desiccator for at least 48 h to remove the remaining isopropanol. The changes in the pore structure were evaluated by mercury intrusion porosimetry (MIP) using a Micrometrics Poresize 9320 mercury intrusion porosimeter (Micromeritics Instrument Corporation, Norcross, GA, USA), assuming a sample–mercury contact angle of 140°. The microstructure of the samples was studied by backscattering electron microscopy (BSEM) on a JEOL JSM6400 (JEOL, Tokyo, Japan) scanning electron microscope. Fragments similar to the ones prepared for MIP were cut, mounted in epoxy resin, abraded with successively finer sandpapers, polished with diamond pastes, cleaned in an ultrasonic bath, dried, coated with carbon, and analyzed in a high-vacuum mode (20kV) in a backscattered electron mode. A semi-quantitative analysis of the chemical composition of the reaction products was conducted by energy dispersive X-ray spectroscopy (EDX) using a Links ISIS EDX (JEOL, Tokyo, Japan) analyzer, collecting at least 40 points from the cementitious matrix per sample; the data were processed using Bruker ESPRIT 2 software (Bruker, Billerica, MA, USA). The mineralogical aspects of samples, including the nanostructural aspect, were studied by X-ray diffraction (XRD), Fourier transform infrared spectroscopy (FTIR), thermogravimetric analysis (TG/DTG), and nuclear magnetic resonance (^31^P MAS NMR). Powder samples were prepared for these measurements from the fragments of the samples previously treated to stop the reaction processes. Specimen fragments were ground to pass through a 63 μm sieve, stirred with isopropanol for 3 min, filtered, and transferred to a desiccator under vacuum until constant weights were achieved. XRD measurements were performed on a Bruker D8 Advance diffractometer (Bruker, Billerica, MA, USA) in a 2θ range of 5–60° with a step size of 0.02° every 0.5 s using CuKα radiation at 40 kV and 30 mA. For the FTIR technique, 1 mg of the sample was mixed with 300 mg of KBr. The acquisition of the FTIR spectra was carried out on an ATIMATT-SON FTIR-TM (Thermo Fisher Scientific, Waltham, MA, USA, 02451) series spectrophotometer at a resolution of 4 cm^−1^ over the 4000–400 cm^−1^ range. Thermogravimetric analysis (TGA) was also carried out, with powdered samples heated from room temperature up to 800 °C at 10 °C min^−1^ in a N_2_ flow (200 cm^3^/min) using a Perkin-Elmer TG analyzer (Perkin-Elmer, Waltham, MA, USA). The ^31^P MAS (magic angle spinning) NMR spectra were recorded at 161.98 MHz in a Bruker AVANCE-400 spectrometer (Bruker, Billerica, MA, USA). The external magnetic field was 9.4 Tesla. All measurements were carried out at 20 °C, and the samples were spun around the magic angle (54°44′ with respect to the magnetic field) at a spinning rate of 10 kHz. The NMR spectra were obtained after a π/2 excitation of 2.85 μs and intervals between successive accumulations of 60 s. The number of accumulations was 40. The ^31^P chemical shift values are given relative to 85% H_3_PO_4_ aqueous solutions.

## 3. Results and Discussion

### 3.1. Mechanical Strengths of MKPC Pastes

**Figure 2:**
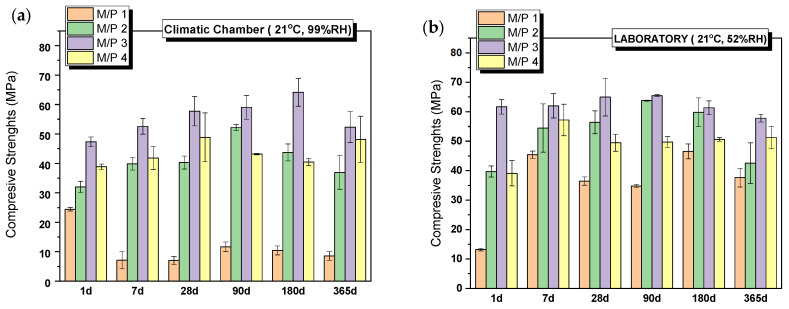
Compressive strengths of MKPCs pastes cured in (**a**) climatic chamber (21 °C, 99% RH) and (**b**) laboratory (21 °C, 52% RH).

All systems set and hardened, developing remarkable mechanical strengths. Figure 2 shows the evolution of compressive strengths with time for systems prepared with different M/P molar ratios (from 1 to 4), cured in the climatic chamber (CC) (99% RH) (Figure 2a) and in the laboratory (LAB) (52%RH) (Figure 2b). The compressive strength increases with the increase in the M/P ratio up to 3, above which the strength slightly decreases. It is well-known that the initial M/P ratio is a key factor for the strength development of MKPCs. According to the literature [31,32], the lower strengths observed in MKPC samples prepared with lower M/P ratios can be attributed to unreacted phosphates, which in high-humidity environments (samples cured in a climatic chamber at 99% RH) leach out of the matrix prematurely, causing changes in the microstructure (a phenomenon also observed in our samples, as explained in Section 3.2). On the other hand, when the M/P ratio is too high, the amount of phosphate is insufficient compared to that of MgO, leading to an inadequate formation of reaction products and ineffective binding of the unreacted particles. As can be observed in Figure 2a,b, the curing conditions, especially the relative humidity, also play an important role in the strength development, especially for the lowest M/P ratio (M/P 1), where the strengths developed in the high-RH (99%) environment are much lower than those obtained in the laboratory. What is more, the strength of the M/P 1 samples decreases substantially with time when cured in the climatic chamber (CC) (from almost 25 MPa at 1 day to below 10 MPa after 7 days). Relative humidity plays an important role in the early reaction stages, promoting the rapid dissolution of phosphates, which induces the formation of efflorescences and changes in the microstructure [33,34,35] (as shown in later results), affecting the strength development.

### 3.2. Characterization of MKPC Pastes

**Figure 3:**
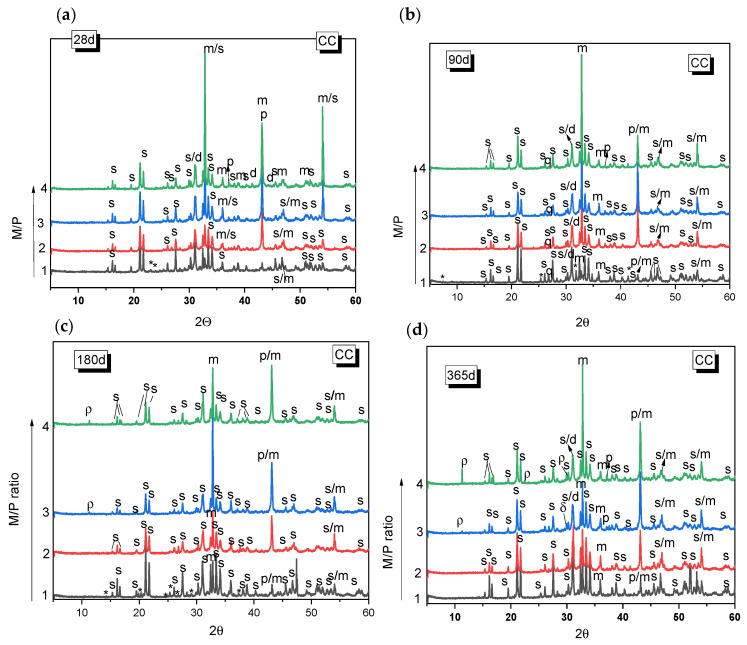
XRD patterns of MKPC samples cured in the laboratory (LAB) (52% RH) after (**a**) 28 d, (**b**) 90 d, (**c**) 180 d, (**d**) 365 d. (Legend: s: K-struvite (MgKPO_4_·6H_2_O-COD: 9011199), p: Periclase (MgO-COD: 9007058), m: magnesite (MgCO_3_-COD: 9000096), d: Dolomite (CaMg(CO_3_)_2_-COD: 1200014), q: Quartz (SiO_2_), *: KMg_2_H(PO_4_)·15H_2_O (PDF 44-0790), ρ: cattite Mg_3_(PO_4_)_2_·22H_2_O).

**Figure 4:**
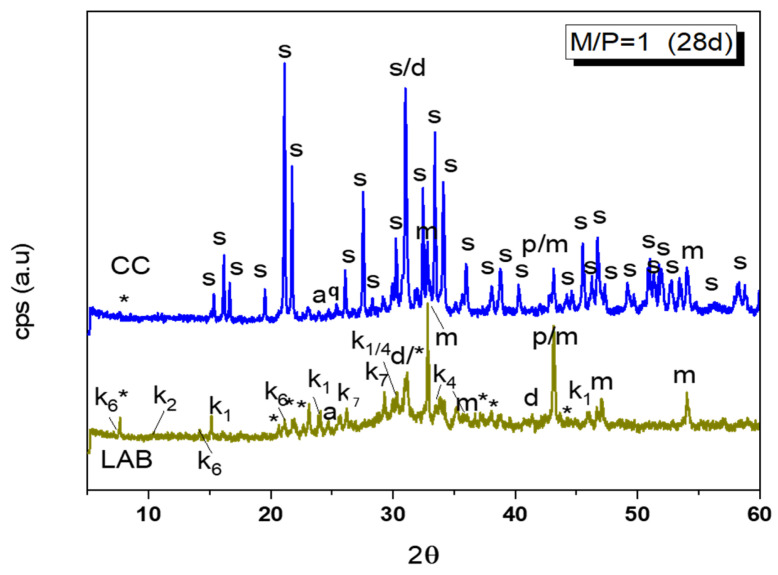
Comparation of XRD patterns of M/P = 1 samples cured in CC (99% RH) and LAB (52% RH) (Legend: s: struvite (MgKPO_4_·6H_2_O-COD: 9011199), *: KMg_2_H(PO_4_)·15H_2_O (PDF 44-0790), p: Periclase (MgO-COD: 9007058), m: magnesite (MgCO_3_-COD: 9000096), d: Dolomite (CaMg(CO_3_)_2_-COD: 1200014), q: Quartz (SiO_2_), a: anhydrite (CaSO_4_) (PDF 37-1496), k_1_: Mg(HPO_2_)_2_(H_2_O)_6_ (PDF 75-0029), k_2_: Mg_3_(PO_4_)_2_·5H_2_O (PDF 35-0329), k_4_: K_4_P_2_O_7_·3H_2_O (PDF 26-1457), k_7_: Mg_2_P_2_O_7_ (PDF 01-0866), k_6_: MgHPO_4_·7H_2_O (phosphorrosslerite) (PDF 46-1267).

Figure 3 and Figure 4 show the XRD patterns for the MKPC paste systems prepared with different M/P ratios (1, 2, 3, and 4), cured either in the climatic chamber or in the laboratory. In all the systems, regardless of the type of curing environment or the M/P ratio, the main reaction product observed was K-struvite. Unreacted periclase was also detected (its peak intensity is higher in the samples with higher M/P ratios). Other phases present in the low-grade MgO (mainly magnesite, dolomite, and quartz) were also identified, but these appear not to significantly participate in hydration or other reactions. The presence of these phases in the original low-grade MgO does not seem to negatively affect the formation of K-struvite. The XRD patterns also indicate the presence of Mg_2_KH(PO_4_)_2_(H_2_O)_15_ in the sample prepared with M/P 1 and cured in the CC, which persists up to 180 days (Figure 3a–c) but disappears by 365 d (Figure 3d). The formation of Mg_2_KH(PO_4_)_2_(H_2_O)_15_ can be observed during the early stage of the reaction stages as an intermediate phase and usually appears at low pH values [36]. The M/P = 1 system has the lowest pH among the systems investigated in the present work due to the highest proportion of KH_2_PO_4_. The presence of Mg_2_KH(PO_4_)_2_(H_2_O)_15_ was not observed in the XRD data samples prepared with M/P ratios of 2, 3, and 4 at the ages studied. In the systems prepared with M/P 3 and 4 and cured in the CC, another intermediate phosphate salt, cattiite, Mg_3_(PO_4_)_2_·22H_2_O, was detected (Figure 3c,d). The intensity of the reflection peaks for this phase increases both with the time of curing and with the M/P ratio. According to the literature [37], the crystallization of K-struvite in an aqueous solution can often be accompanied by the co-precipitation of cattiite based on Reaction 2.

3Mg^2+^ + 2HPO_4_^2−^ + 22H_2_O → Mg_3_(PO_4_)_2_·22H_2_O + 2H^+^. (R2)

As the pH value increases (with the increase in the M/P ratio), K-struvite and cattiite are stabilized [36]. It should be noted that despite working with high M/P ratios and a relative humidity of >99%, no crystalline brucite (Mg(OH)_2_) was detected by XRD. The absence of brucite could suggest that the system reacts with phosphates to form cattiite according to the Reaction 3:

3Mg(OH)_2_ + 2KH_2_PO_4_+ 18H_2_O → Mg_3_(PO_4_)_2_·22H_2_O + 2KOH (R3)

The samples cured in the laboratory (52% RH) showed generally similar features as those cured in the higher relative humidity and also showed K-struvite as the main reaction product except for the systems where M/P = 1, where several types of phosphates salts were equally identified.

A detailed XRD pattern of this sample is shown in Figure 4, where different types of phosphates can be identified: unreacted KH_2_PO_4_, MgP_2_O_7_·XH_2_O, MgH_2_P_2_O_7_, K_4_P_2_O_7_·3H_2_O, MgHPO_4_·7H_2_O, MgHPO_4_·3H_2_O (newberyite), and KMg_2_(H(PO_4_)·15H_2_O. The XRD pattern of this sample appears, when compared with the one cured at the higher humidity, much less crystalline, and a broad hump is observable in 28–36 2θ, indicating the potential formation of an amorphous phosphate phase.

Based on the previous results, selected samples were chosen for further analysis.

**Figure 5:**
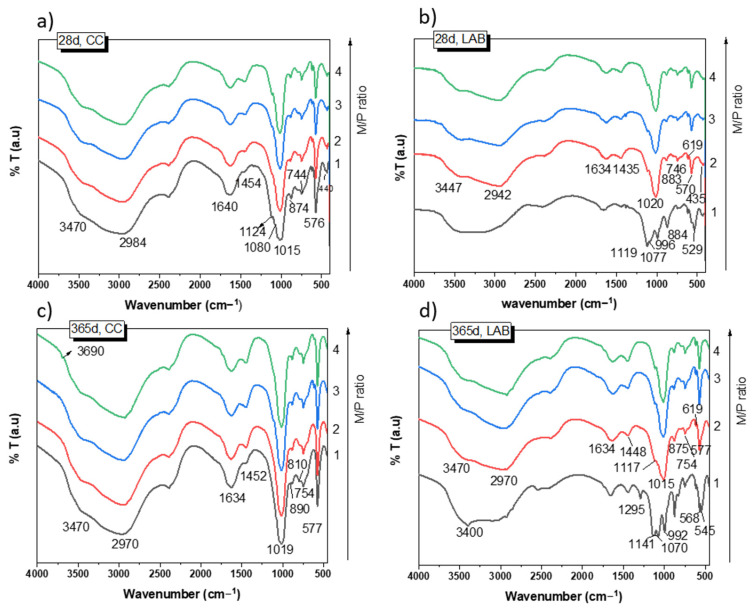
FTIR spectra of MKPC: (**a**) 28 d, CC; (**b**) 28 d, LAB; (**c**) 365 d, CC; (**d**) 365 d, LAB.

Figure 5 shows the FTIR spectra of the samples cured for 28 and 365 days. After 28 days of curing at 99% RH (Figure 5a), the spectra of the samples show practically the same absorption bands for all M/P ratios. They are different from those detected in the raw materials (see Figure 1b,c). It should be noted that the typical bands located at 1300 cm^−1^ (υ as P=O), 1100 (υ as P-O-P), and 910 cm^−1^ (υ as P-O-P) detected in the phosphate source (KH_2_PO_4_, see Figure 1) completely disappeared in all the MKPC spectra, indicating its complete dissolution. The assignment of the bands is shown in Table 2. Most of these bands are associated with different vibration modes in K-struvite [37,38,39,40]. Some minor signals were also observed that were associated with unreacted secondary phases (magnesite, dolomite, anhydrite, and quartz) present in the low-grade MgO. No significant changes were detected in these spectra after 365 days of curing (Figure 5c), with the exception of a small shoulder appearing at 3690 cm^−1^ attributed to υ as H-O-H [39] of a small proportion of brucite (not detected by XRD). This shoulder was observed only in samples prepared with an M/P ratio of 3 and 4.

Samples cured at low RH (52%) show absorption bands (Figure 5b,d) at the same wave numbers as those cured at high RH (Figure 5a) when the M/P is 2, 3, and 4; most of the bands are associated with the precipitation of K-struvite. However, the MKPC system with an M/P ratio of 1 indicated a very different FTIR spectrum, with a new group of bands and shoulders located around 1295 cm^−1^, 1120 cm^−1^, and 992 cm^−1^ (Figure 5b,c), which are assigned to υ (OH) modes (in-plane POH bending vibrations) and υ as P-O and P-OH bending, respectively [27], most likely suggesting the precipitation of several phosphate salts different from the K-struvite detected by XRD. The assignment of these bands to specific phases is very challenging due to the possible overlapping of the different vibration modes (P-O) of the different phosphate compounds.

**Figure 6:**
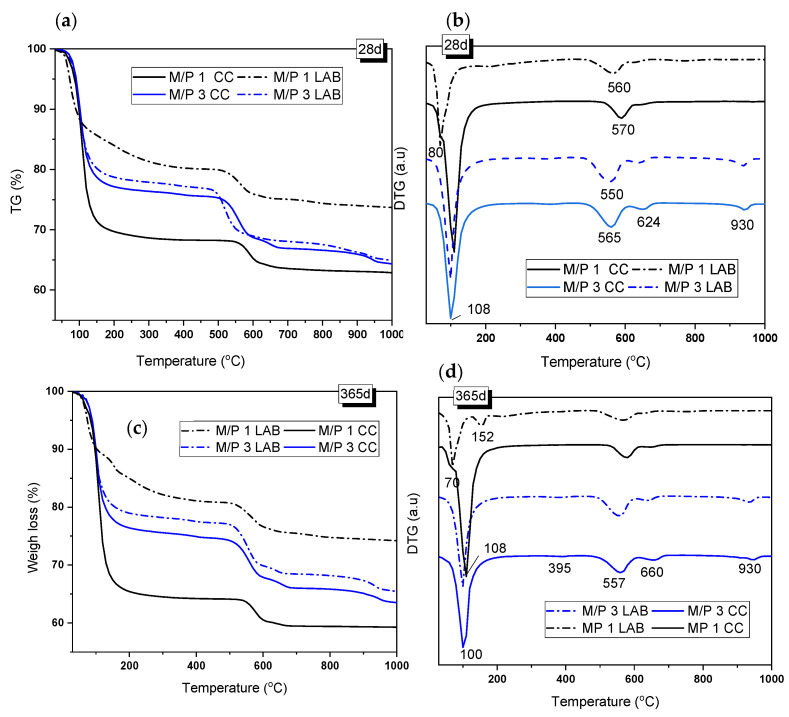
(**a**,**c**) TG and (**b**,**d**) DTG curves of samples prepared with M/P ratios of 1 and 3 and cured in the LAB and in the CC after 28 and 365 days.

Figure 6 shows the TG/DTG curves of the samples with M/P ratios of 1 and 3 after 28 and 365 days of curing. The TG and DTG curves show three main weight-loss events: one in the range of 50–200 °C, with the second and third at 480–650 °C and above 900 °C, respectively. The first event was associated with the loss of water from hydrated phosphate-based products [36,38,41], while the second and third events corresponded to the decarbonization of magnesite (MgCO_3_ → MgO +CO_2_) (around 560 °C) and the decarbonization of dolomite, which takes place in two steps: (i) MgCa(CO_3_)_2_ → MgO + CaCO_3_) (first peak at around 630–660 °C) and (ii) CaCO_3_ → CaO+ CO_2_ (second peak at around 930 °C) [39,41]. Both carbonates were identified as secondary phases in the low-grade MgO. K-struvite has been known to have loss of water between 90 and 200 °C [39] (see Reaction 4).

MgKPO_4_·6H_2_O → MgKPO_4_ + 6 H_2_O↑ (R4)

After 28 days of curing, the weight loss associated with this phase was 23.44% and 21.44% for the M/P 3 samples cured in the CC and in the LAB, respectively, indicating that in this particular case, the RH used during the curing did not affect the amount of this phase that was precipitated. However, in the case of the M/P ratio of 1, TG/DTG curves were significantly different from the M/P 3 system and indicated the influence of the type of curing; the sample cured at a high RH showed a DTG peak at around 108 °C (Figure 6b) (corresponding weight loss of 30.6%) that can be attributed to the loss of water in K-struvite. However, for the sample cured in the laboratory, the DTG peak appeared shifted towards a lower temperature (80 °C), and the weight loss was significantly smaller (~18%), confirming that in this particular sample, the dominant phase was not K-struvite but other phosphate materials, which was suggested by the XRD and FTIR data (Figure 4 and Figure 5).

According to the literature [40], the weight-loss event that occurred at ~70 °C could represent the dehydration of Mg_2_KH(PO_4_)_2_·15H_2_O (Reaction 5), which is one of the phases identified in the M/P 1 sample cured in the laboratory. The presence of the same phase can also be confirmed in the systems cured in the CC, with a small shoulder that also appears at this temperature (~80 °C).

Mg_2_KH(PO_4_)_2_·15H_2_O → Mg_2_KHP O_4_ + 15H_2_O (R5)

After 365 days of curing, the TG/DTG curves were very similar to those at 28 days but with some differences. The M/P 3 systems, cured both in the CC and in the LAB, present a small peak located at 395 °C that could correspond to the dehydroxylation of brucite (Mg(OH)_2_ → MgO + H_2_O) [41], which was not identified by XRD but was observed by FTIR (see Figure 5). This peak corresponds to a weight loss of 0.6–0.7%, indicating that it is a minor component in the mixture. On the other hand, in the M/P 1 system cured in the laboratory, the signal that appears around 70–80 °C was still observed, but an additional signal appeared at 152 °C, which, according to the literature, could correspond to the dehydration of MgKPO_4_.xH_2_O [42]. This phase, also identified by XRD, usually presents water loss in the range of 90 to 250 °C. For the samples prepared with M/P 3, the mass loss corresponding to the water loss of K-struvite remained constant with the curing time, which could indicate that after 28 days, the amount of this phase remains practically constant.

**Figure 7:**
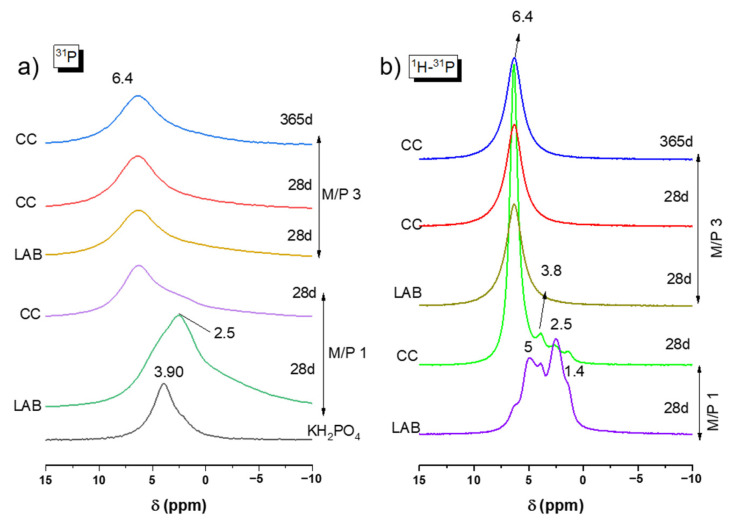
(**a**) ^31^P MAS NMR and (**b**) CP 1H-^31^P of KH_2_PO_4_ and MKPC samples prepared with M/P 1 and 3.

The ^31^P MAS NMR spectra for the precursor (KH_2_PO_4_) and MKPC samples prepared with M/P ratios of 1 and 3 are plotted in Figure 7a. KH_2_PO_4_ exhibits a chemical shift peak located around +3.8 ppm associated with the H_2_PO_4_^−^ environment. This observation was also reported in previous studies by Scrimgeour et al. and Zhang G et al. [43,44]. The samples prepared with M/P 1 exhibit a significant difference compared to those with M/P 3, especially the one prepared in the laboratory. A peak is seen around +2.5 ppm, which could be attributed to secondary phosphate phases different from K-struvite, such as Mg_2_KH(PO_4_)_2_·15H_2_O, as detected in the XRD analysis. This phosphate phase has also been reported in the literature [44,45,46,47]. For MKPC prepared with M/P 3, only one chemical shift peak was observed at +6.4 ppm for all samples at any curing ages both in the climatic chamber (CC) and in the laboratory (LAB) environments. This peak corresponds to the PO_4_ units in K-struvite (MgKPO_4_·6H_2_O) [39,40]. Apparently, no other resonances that could be attributed to intermediate phosphate phases were observed.

Figure 7b presents the 1H-^31^P CPMAS NMR spectra for all samples. In each spectrum, a peak appears at +6.4 ppm, which is attributed to K-struvite. However, in the M/P 1 LAB 28 d sample, this phase is not dominant, and several shoulders are observed around +5 ppm, which could indicate the presence of the phase Mg_3_(PO_4_)_2_.5H_2_O (close to the position of bobierrite (Mg_3_(PO_4_)_2_·8(H_2_O)) (+4.6 ppm); at +3.8 ppm for remaining KH_2_PO_4_; at +2.5 ppm associated with Mg_2_KH(PO_4_)_2_·15H_2_O; and at +1.4 ppm, which could be assigned to MgH(PO_4_)·7H_2_O, all of which were identified by XRD (Figure 4). The same formulation cured in the climatic chamber show a peak again at +6.4 ppm (K-struvite) as the main resonance, but there were still some small shoulders present at the same position (+3.8 ppm, +2.5 ppm, and +1.4 ppm).

Although only the presence of K-struvite was predominantly identified in the ^31^P NMR data for most samples, the broad asymmetrical nature y of the ^31^P NMR signals suggest that the spectrum is probably the result of contributions from multiple overlapping components.

**Figure 8:**
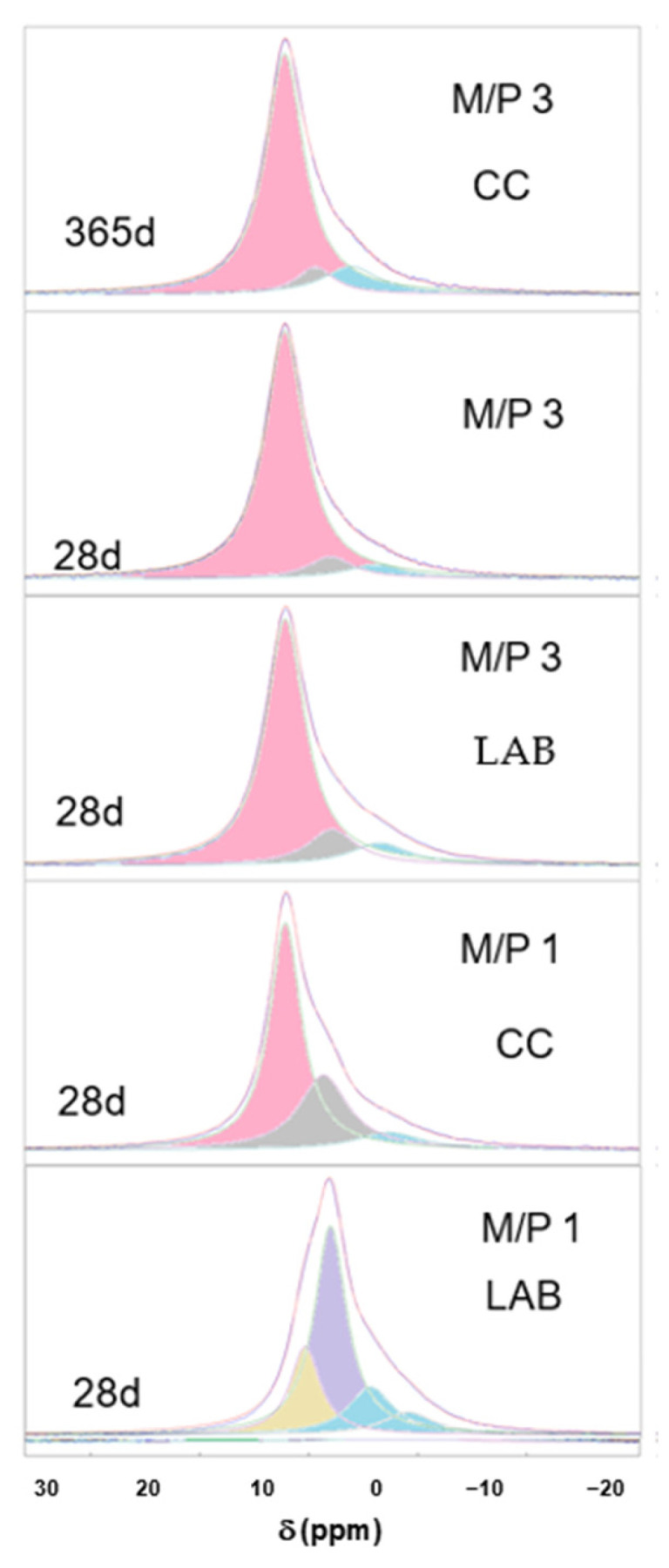
Deconvoluted ^31^P spectra of MKPC samples (pink components correspond to K-struvite, blue components correspond to amorphous phases, and the rest are associated with the secondary phosphate phases).

Figure 8 shows the deconvoluted ^31^P spectra of the samples, which are also summarized in Table 3. It can be seen that in most of the spectra (with the exception of sample M/P 1 LAB), the component appearing at +6.4 ppm corresponds to K-struvite, with other small contributions appearing in the range between +0.5 and −5 ppm that are associated with an amorphous phase previously identified in the literature [40,44,45,46], which precipitates together with K-struvite.

In the deconvoluted spectrum of the M/P1 LAB 28 d sample, the signal at −0.63 ppm could correspond to an amorphous MgHPO_4_ phase [44,45]; and the one appearing at −4.64 ppm corresponds to Mg_2_P_2_O_7_ pyrophosphates [33,44], also detected by XRD while the components appearing at +5 ppm and +2.4 ppm are the contributions of the phases Mg_3_(PO_4_)_2_·5H_2_O and Mg_2_KH(PO_4_)_2_·15H_2_O, respectively [44,46]. 

### 3.3. Changes in the Microstructure of MKPC Pastes

**Figure 9:**
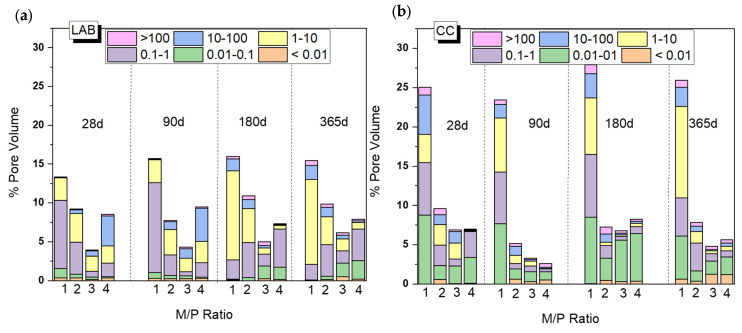
Total porosity (%) and pore size distribution of MKPC pastes cured in (**a**) a climatic chamber (99% RH) and (**b**) a laboratory (52% RH).

Figure 9 shows changes in the total porosity and pore size distribution with curing time (from 28 d to 365 d) depending on the M/P ratio and curing environment (CC and LAB). Both the M/P ratio and the type of curing condition have a clear impact on the microstructure, which explains the mechanical strengths shown in Figure 2. There is a decrease in the total porosity with the increase in the M/P ratio up to M/P = 3, which rises at M/P = 4, especially in the laboratory condition (Figure 9b). Samples cured in the CC (Figure 9a) show higher total porosities than those cured in the laboratory, and this is much more notable with an M/P ratio of 1, with which the total porosity exceeds 25%. The higher RH during the curing (99% RH) promotes the fast dissolution of phosphates, which is reflected in the increase in the total porosity and, therefore, a notable decline in the strengths (Figure 2). Pore size distribution was also different depending on the type of curing environment; the more representative pores in samples cured in the laboratory were in the ranges of 1–10 μm and 0.1–1 μm, while bigger pores (≥10 microns) were observed in systems cured at higher relative humidity.

Based on the previous results (Figure 9), the system corresponding to an M/P ratio of 3 presented higher mechanical strengths, the lowest porosity, and the highest mineralogical stability (the main reaction product is K-struvite, which does not show changes up to 365 days based on the XRD and FTIR). On the contrary, the systems prepared with the lowest M/P ratio of 1 showed the least strength development, the highest porosity (especially the system cured in the CC), and the presence of several types of phosphates (especially the samples cured in the laboratory).

**Figure 10:**
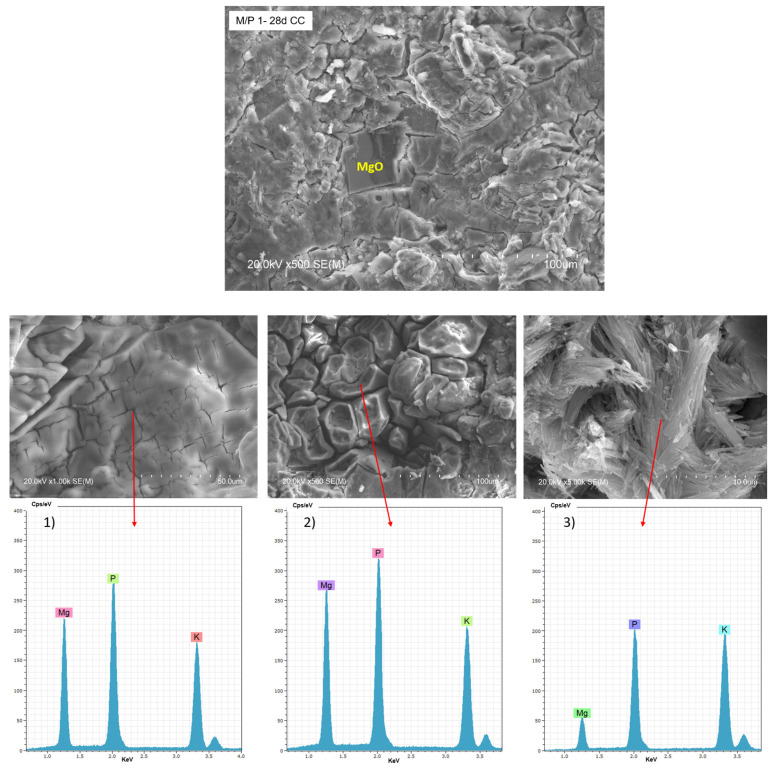
BSEM micrographs and EDX (1, 2 and 3) analysis of the M/P 1 sample after 28 days of curing in the climatic chamber CC (99% RH) at different magnification.

Figure 10 shows BSEM/EDX data of the M/P 1 sample after 28 days of curing in the climatic chamber (CC). Unreacted particles of MgO can be observed, surrounded by a matrix based on Mg, P, and K (EDX 1), attributed to K-struvite. Different types of morphologies are observable in the matrix: a bladed prismatic shape with a composition matching K-struvite (EDX 2) and acicular rods with a chemical composition richer in P (EDX 3), which corresponding to other types of phosphates and consistent with NMR data (Figure 7). Both types of morphologies were already reported in the literature [48,49].

**Figure 11:**
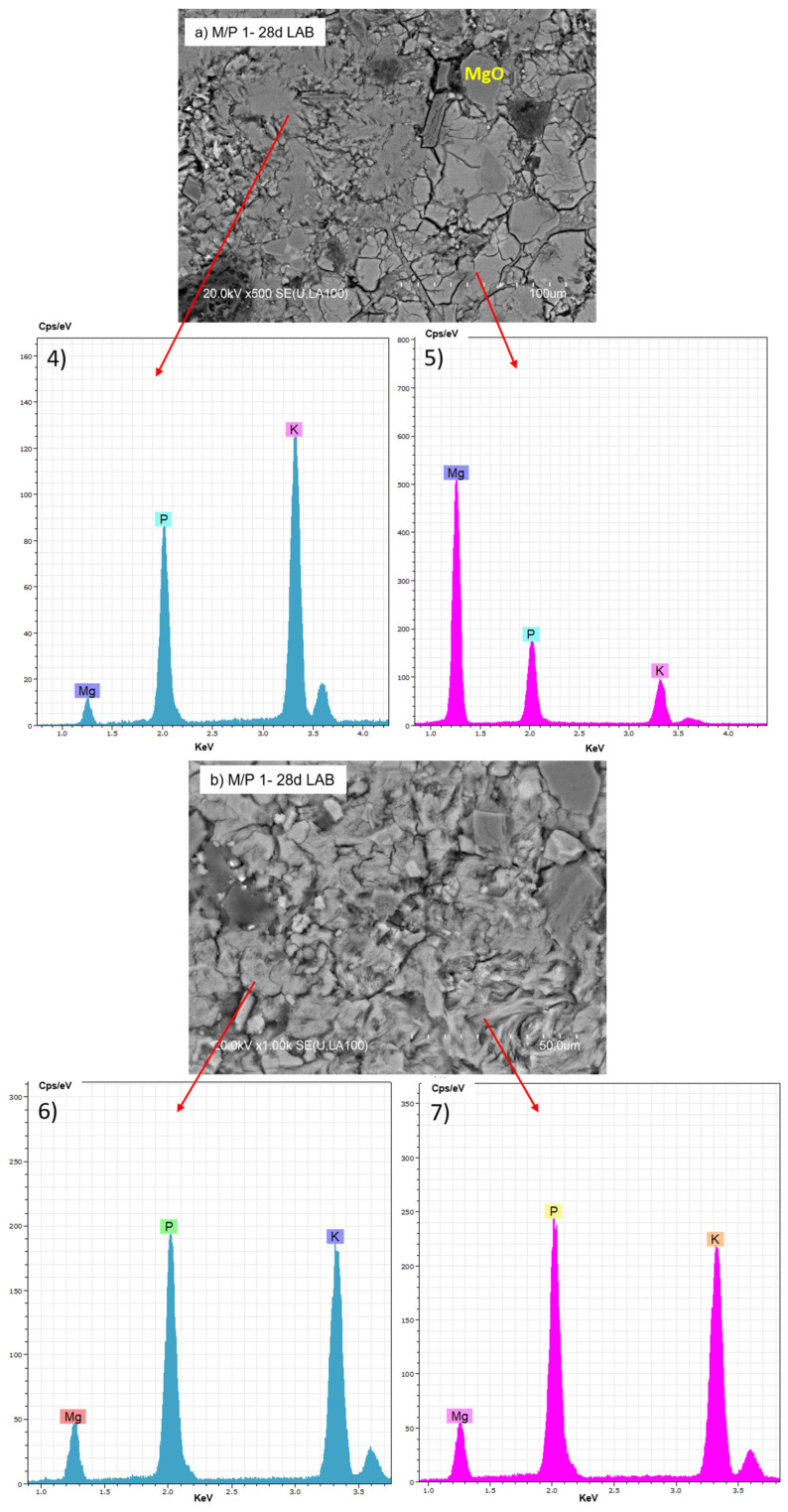
BSEM micrographs and EDX (4, 5, 6 and 7) analysis of M/P 1 sample after 28 days of curing in the laboratory: (**a**) 500× magnification; (**b**) 1000× magnification.

The BSEM micrographs of the M/P 1 sample cured at lower RH (LAB) are shown in Figure 11. Several morphologies can be distinguished: some appear more globular, while others are more fibrillar (Figure 11b). Both exhibit a chemical composition based on K, P, and Mg but with different M/P and K/P ratios, suggesting the presence of different secondary phosphate phases identified by XRD and NMR.

**Figure 12:**
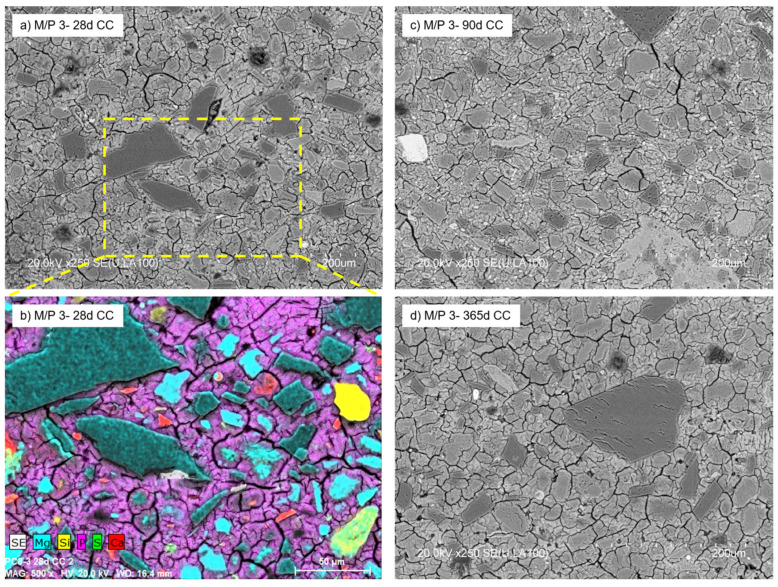
BSEM micrographs of M/P 3 samples cured in CC at (**a**) 28 days; (**b**) EDX mapping at 28 days, (**c**) 90 days, and (**d**) 365 days.

With a further increase in the M/P ratio to 3, the formed products no longer have a definite shape but have a less condensed microstructure with a large number of voids observed in the matrix (Figure 12). The micrograph (Figure 12a) shows the presence of a significant number of unreacted particles of periclase surrounded by a matrix (mainly K-struvite). The secondary crystalline phases detected in the low-grade MgO (quartz, magnesite, dolomite, and anhydrite) were also detected by mapping (Figure 12b). There were no significant changes in the microstructure with age (90 and 365 d) (Figure 12c,d), which is in agreement with the results shown by MIP.

**Figure 13:**
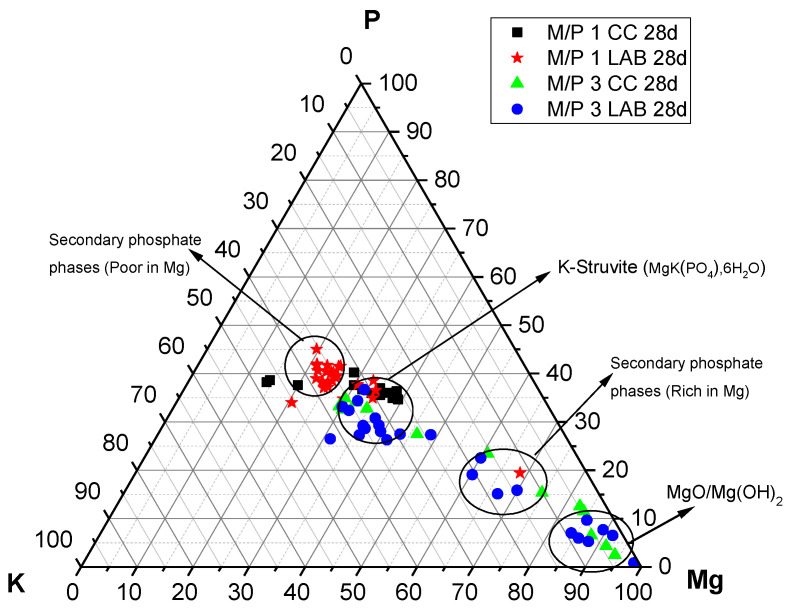
EDX analysis plotted on a ternary diagram (P-Mg-K molar) of MKPC cements prepared with M/P ratios of 1 and 3 (CC and LAB) at 28 days.

The P, Mg, and K compositions of different regions in the 28-day samples examined by EDX are plotted in Figure 13. It is interesting to see that the compositions determined by the EDX analysis are all distributed along the yellow line, signifying P:K = 1:1 regardless of the M/P ratio and curing environment. On the other hand, the composition of Mg varies, and the M/P 1 system indicated that the compositional distribution of Mg in a relatively narrow range, while the M/P 3 system had a wider compositional distribution for Mg. The curing condition (CC or LAB) appears to be less impactful in terms of the compositional distribution of Mg in the product phases.

**Figure 14:**
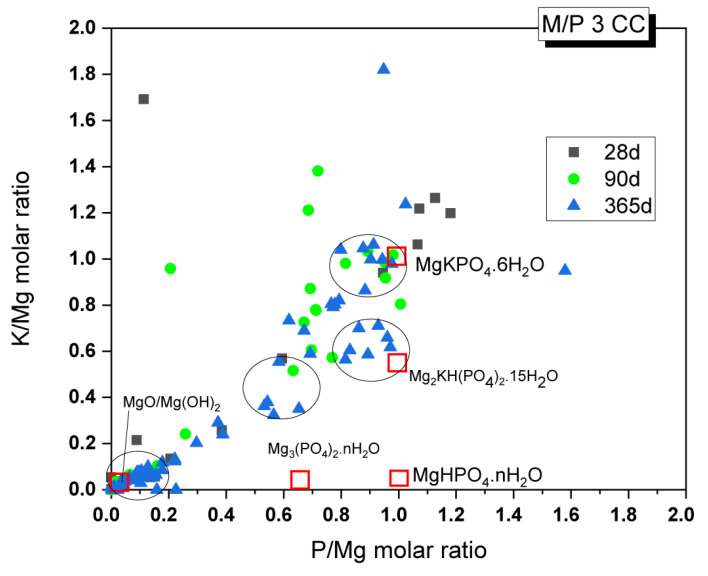
EDX analysis results of the M/P 3 samples cured in the CC after 28, 90, and 365 days.

Changes in the chemical composition of the cementitious matrix over time were also analyzed. The obtained K/Mg molar ratio is plotted against the P/Mg molar ratio in Figure 14. The figure also shows the theoretical K/Mg and P/Mg ratios of the phases commonly detected in MKPC pastes: MgO, Mg(OH)_2_, MgHPO_4_·nH_2_O, Mg_3_(PO_4_)_2_·22H_2_O, Mg_2_KH(PO_4_)_2_·15H_2_O, and K-struvite (MgKPO_4_·6H_2_O). Four clusters of composition can be easily distinguished: (I) compositions close to the theoretical K-struvite (P/Mg 1 and K/Mg 1) with younger samples showing higher P/M ratios; (II) a cluster close to the theoretical Mg_2_KH(PO_4_)_2_·15H_2_O but with higher P/Mg molar ratios; (III) a cluster close to the chemical composition of cattiite, Mg_3_(PO_4_)_2_·22H_2_O, but with some K in its composition; and (IV) a cluster with a chemical composition close to MgO or Mg(OH)_2_.

Considering that the main reaction phase observed in the present study was K-struvite, cluster (I) corresponds to this phase. According to the literature [31], the higher P/Mg ratio (with respect to the theoretical P/Mg = 1) observed in the younger samples could be explained by several reasons: (i) the initial formulation of the system has a higher value than the theoretical P/Mg ratio of 1, (ii) phosphate anions may have been adsorbed onto the crystal surface, affecting the EDX analysis results, or (iii) other phosphates were formed with the impurities originally present in the unreacted magnesia. Although intermediate phases, such as Mg_2_KH(PO_4_)_2_·15H_2_O, were not positively identified by XRD, there is a cluster of composition close to these phases, especially in samples after 365 days, which contradicts the information in the literature, where the precipitation of these phases is usually associated with early ages. The absence of crystalline phases, with the exception of K-struvite, led us to think that cluster (II) could correspond with an amorphous phase with a chemical composition close to this phase (P/Mg ~1). This would be in line with the results observed by ^31^P NMR, where a component corresponding to an amorphous phase was identified in the deconvoluted spectra. Cluster (III) would correspond with phases such as Mg_3_(PO_4_)_2_·22H_2_O (observed by XRD in a minor proportion, especially in the older samples), which adsorbed some K ions on the surface, resulting in the small proportion of K detected. Regarding cluster (IV), which is close to MgO and Mg(OH)_2_, the presence of brucite was detected in small amounts, especially in the older samples, as confirmed by FTIR and TG/DTG. This finding is consistent with the presence of the Mg(OH)₂ phase, but it primarily indicates the presence of phosphate on the surface of magnesium oxide, a phenomenon also observed by other researchers [27].

### 3.4. Changes in the Volume Stability of MKPC Mortars

**Figure 15:**
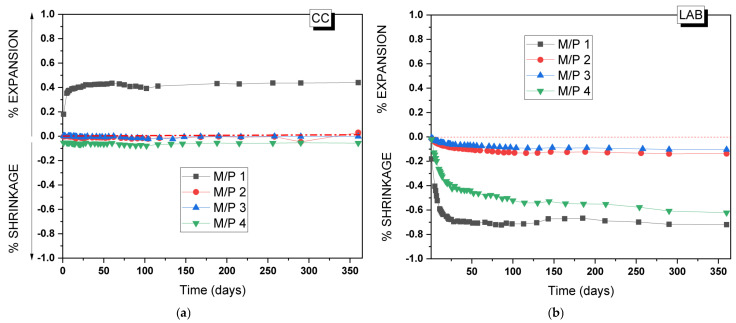
Volume changes of MKPC mortars stored in (**a**) the climatic chamber (21 °C, 99% RH) and (**b**) the laboratory (21 °C, 52% RH).

To assess the practical application of the MKPC developed in the present study, the volume stability of the MKPC mortars was analyzed for up to 365 days. Figure 15a,b show, respectively, changes in the volume of the mortars prepared with different M/P ratios when stored in the climatic chamber (99% RH, 21 °C) and in the laboratory (52% RH, 21 °C). As can be seen, volume stability is highly dependent on both the M/P ratio and the relative humidity of the storage conditions [34,50]. MKPC mortars stored at 99% RH (Figure 15a) show a good volume stability except for the lowest M/P ratio of 1, where a clear expansion (up to 0.4%) was observed, the maximum of which was reached at 28 days. This type of expansion was reported in the literature [34] in a high relative humidity and low M/P ratios. Since no brucite was identified after 28 days in the M/P 1 samples, the observed expansion cannot be attributed to the formation of brucite. Instead, it may be linked to the intermediate phosphate phase (Mg_2_KH(PO_4_)_2_(H_2_O)_15_), which was observed in the XRD (Figure 3) and was characterized by its large, plate-like crystals [51]. These large plate-like crystals may contribute to the observed expansion. The excessive formation of this product in the hardened paste increases internal stresses at the microstructural level. This behavior also justifies the decrease in strength in this particular systems over time (Figure 2a). On the contrary, samples stored in the laboratory showed shrinkage (Figure 15b), most likely attributed to the drying shrinkage caused by the evaporation of water at low RH. The M/P 1 and 4 systems were the most unstable; however, samples prepared with M/P ratios of 2 and 3 showed a good volume stability under the same condition. Pores affecting shrinkage are generally gel pores (diameter < 10 nm) [40] and transitional pores (diameter 10–100 nm) [41,42]. The pore size distribution of the paste samples (Figure 9) could justify this behavior. These data also agree with the mechanical strengths of the paste samples, where the best results were obtained for the systems prepared with M/P ratios of 2 and 3.

## 4. Conclusions

In the present study, the mineralogy, microstructure, and nanostructure of MKPC cements were examined to explore the applicability of a low-grade MgO, in particular to be used as a potential encapsulation matrix for low and intermediate nuclear waste. Based on the obtained results, the main conclusions can be drawn as follows:

- A low-grade MgO (~58% MgO) can be effectively used to produce MKPC cements. The presence of secondary minor phases (such as magnesite, dolomite, anhydrite, and quartz) does not affect the formation of the main reaction product, K-struvite crystals.
- The M/P molar ratio and relative humidity are key parameters to be considered in the design of MKPC cements. At a lower M/P ratio (M/P 1), the high phosphate content can induce the formation of secondary phases different from the K-struvite. Additionally, elevated relative humidity promotes efflorescence formation, altering the microstructure and porosity, which in turn affects the mechanical strength.
- For samples prepared with M/P 2, 3, and 4, K-struvite is the primary reaction product regardless of relative humidity conditions. However, the formation of secondary crystalline phases, such as Mg_2_KH(PO_4_)_2_·15H_2_O and Mg_3_(PO_4_)_2_·22H_2_O, is favored in samples cured at high relative humidity. Additionally, the co-precipitation of an amorphous phase composed of Mg, P, and K with little to no water content was confirmed under both curing conditions.
- Systems prepared with an M/P ratio of 3 achieved compressive strengths exceeding 60 MPa under both relative humidity conditions after 28 days. Additionally, the microstructure (total porosity and pore size distribution), the stable mineralogy (the mayor reaction product is K-struvite), and the good volume stability, make this formulation a potential candidate for the encapsulation of LL and IL nuclear waste.

## Figures and Tables

**Table 1:** Formulation of the pastes (1 × 1 × 6 cm^3^) and mortars (2.5 × 2.5 × 28.5 cm^3^) prepared.

	M/P Molar Ratio *	W/B Ratio **	LG-MgO (g)	KH_2_PO_4_ (g)	Boric Acid (g)	Sand (g)
Pastes (1 × 1 × 6 cm^3^)	1	0.25	44.76	87.40	2.73	-
	2	0.25	43.00	41.96	1.67	-
	3	0.25	51.60	33.75	1.59	-
	4	0.25	51.60	25.31	1.38	-
Mortars (2.5 × 2.5 × 28.5 cm^3^)	1	0.30	350	602.97	15.63	952.97
	2	0.30	500	430.69	13.71	930.69
	3	0.30	600	344.55	13.01	944.55
	4	0.30	600	294.47	894.47	12.86

* M = MgO, P = KH_2_PO_4_, ** water/binder ratio (binder = low-grade MgO + KH_2_PO_4_) (in weight). (Considering the final application of these MKPC systems (nuclear waste encapsulation), all systems were prepared with the minimum amounts of water required to have a good workability).

**Table 2:** Assignment of the FTIR bands in MKPC systems.

Wave Number (cm^−1^)	Assignment	Ref.
~3690	υ_as_ H-O-H (brucite, Mg(OH)_2_)	[39,40]
~3426, 2956 cm^−1^	υ_as_ H-O-H cluster of water molecules of crystallization	[27]
~2393 cm^−1^	υ_as_ HOH cluster of water molecules of crystallization	[27]
~1260 cm^−1^	δ OH deformation vibration of water/symmetric stretch of (P=O) bonds, υs(P=O)	[27]
~1450–1440 cm^−1^	υ_as_ CO (CO_3_^2−^)/magnesite–dolomite	[27,40]
~1120 cm^−1^	υ_as_ POP (PO_3_^−^), υ_as_ S-O (anhydrite), υ_as_ Si-O (quartz)	[27,39,40]
~1015–1020 cm^−1^	υ_as_ P-O (PO_4_) K-struvite	[37,38,40]
~883 cm^−1^	Wagging modes of coordinated water (K-struvite)	[37,38,40]
~784 cm^−1^	υ_as_ P-O K-struvite	[37,38,40]
~746–754 cm^−1^	υ C-O magnesite	[38,40]
~571–575 cm^−1^	δ P-O-P, K-struvite/δ P-O-P (PO_4_^3−^)	[37,38,40]
~435–440 cm^−1^	δ P-O-P, K-struvite and/or δ(P=O)	[37,38,40]

**Table 3:** δ ppm (area %) of the deconvoluted ^31^P NMR spectra of MKPC samples.

	δ ppm (Area %)			
M/P 3 CC 365 d	+6.45 ppm (78.76%)	+3.68 ppm (8.44%)	+0.46 ppm (12.80%)	
M/P 3 CC 28 d	+6.44 (85.12%)	+2.28 ppm (8.30%)	+0.40 ppm (6.59%)	
M/P 3 LAB 28 d	+6.39 ppm (76.46%)	+2.16 ppm (13.56%)	−1.16 ppm (9.98%)	
M/P 1 CC 28 d	+6.38 ppm (60.14)	+2.63 ppm (30.60%)	−2.4 ppm (9.26%)	
M/P 1 LAB 28 d	+5 ppm (53.65%)	+2.4 ppm (20.46%)	−0.63 ppm (16.88%)	−4.64 ppm (9.01%)

## Data Availability

The original contributions presented in the study are included in the article, further inquiries can be directed to the corresponding author.
